# Diagnosis, Pathophysiology and Management of Microvascular Dysfunction in Diabetes Mellitus

**DOI:** 10.3390/diagnostics14242830

**Published:** 2024-12-16

**Authors:** Yih-Kuen Jan, Nicolas Kelhofer, Tony Tu, Owaise Mansuri, Kingsley Onyemere, Shruti Dave, Suguna Pappu

**Affiliations:** 1 Department of Health and Kinesiology, University of Illinois at Urbana-Champaign, Urbana, IL 61801, USA; 2 Carle Illinois College of Medicine, University of Illinois at Urbana-Champaign, Urbana, IL 61801, USA; nck7@illinois.edu (N.K.); tonytu2@illinois.edu (T.T.); 3 Department of Endocrinology, Carle Foundation Hospital, Urbana, IL 61801, USA; owaise.mansuri@carle.com (O.M.); kingsley.onyemere@carle.com (K.O.); shruti.dave@carle.com (S.D.); 4 Department of Neurosurgery, Carle Foundation Hospital, Urbana, IL 61801, USA; spappu@illinois.edu

**Keywords:** diabetic foot ulcers, diabetic peripheral neuropathy, endothelial, exercise, laser Doppler, near-infrared spectroscopy, oximetry, vasomotion

## Abstract

Microcirculation is an essential system that regulates oxygen and nutrients to cells and tissues in response to various environmental stimuli and pathophysiological conditions. Diabetes mellitus can cause microvascular complications including nephropathy, neuropathy, and retinopathy. The pathogenesis of microvascular dysfunction in diabetes is associated with hyperglycemia and the result of an interplay of various factors. Research studies have demonstrated that functional microvascular dysfunction appears much earlier than structural alterations in vasculature in diabetes. This finding of the progression from microvascular dysfunction to macrovascular disease establishes a foundation for the screening and early diagnosis of diabetes by assessing the microvascular function. This comprehensive review discusses technologies (laser Doppler, transcutaneous oximetry, infrared thermography and near-infrared spectroscopy) with computational methods (linear (time and frequency domains), nonlinear and machine learning approaches) for diagnosing microvascular dysfunction in diabetes. Pathophysiological changes of microvascular dysfunction leading to impaired vasomotion and blood flow oscillations in diabetes are reviewed. Recent findings in managing microvascular dysfunction using lifestyle modifications and force-based modulations are evaluated. A consensus endorsed by the American Diabetes Association has been reached that an effective exercise program would greatly slow down the progression of microvascular dysfunction and its impact on diabetic foot ulcers, muscle fatigue and weakness and peripheral neuropathy. However, it is imperative to determine the dose–response relationship of exercise and microvascular responses in patients with diabetes. Research studies have demonstrated that local vibration and whole-body vibration can improve microcirculation in various pathological conditions, including diabetes. Due to the complex nature of microvascular regulation, various computational methods have been developed to shed light on the influence of diabetes on microvascular dysfunction. This comprehensive review will contribute to the diagnosis and management of microvascular dysfunction in diabetes.

## 1. Introduction

Diabetes mellitus (DM) can cause microvascular complications, including nephropathy, neuropathy, and retinopathy [1,2]. Research studies have demonstrated that microvascular dysfunction appears much earlier than structural alterations in vasculature in diabetes. This finding of the progression from microvascular dysfunction to macrovascular disease establishes a foundation for the screening and early diagnosis of diabetes by assessing microvascular function [2,3,4]. The early diagnosis of microvascular dysfunction enables clinicians to monitor the progression from pre-diabetes to diabetes and provide in a timely way preventive interventions for managing various microvascular complications.

Microcirculation is the system that delivers oxygen and nutrients to meet the need of local cells and tissues in response to various environmental stimuli and pathophysiological conditions. The study of the structure, function, regulation and remodeling of microcirculation has been playing a critical role in understanding microvascular complications and cardiometabolic diseases [1,5,6]. Diabetes can affect the microvasculature in multiple organs and tissues, including the eye, peripheral nervous system, skin and skeletal muscles. The pathogenesis of microvascular dysfunction in diabetes is associated with hyperglycemia and is the result of an interplay of various factors [1,4]. Although diabetes can cause various degrees of microvascular impairment in organs, it is generally accepted that cutaneous microcirculation is the most accessible site for the early diagnosis of diabetes-related microvascular dysfunction [1,5,6]. As a result, cutaneous microcirculation has been studied to better understand the effect of diabetes on microvascular dysfunction and cardiometabolic disease [7]. Various preventive and rehabilitative interventions have been proposed to manage microvascular dysfunction in diabetes. The American Diabetes Association (ADA) indicates that lifestyle modifications and physical activity are the most essential components of diabetes management as well as the prevention of diabetic complications [8,9,10]. Recent advances in optical and imaging technologies with advanced computational methods have provided new windows to investigate diabetes-associated microvascular dysfunction and its role in secondary conditions and complications in prediabetes and diabetes. This comprehensive review discusses recent advances in computational methods for screening and diagnosing microvascular dysfunction that are not yet widely adopted into the clinical practice of diabetes management. This review is organized into three sections including recent advances in the diagnosis, pathophysiology findings and treatment of microvascular dysfunction in diabetes. The included studies are studies of new technologies and methods used to screen and diagnose microvascular dysfunction and trials of new interventions used to improve microvascular dysfunction in diabetes. The purpose of this review is to discuss principles of new technologies for assessing microvascular dysfunction, evaluate new findings of the pathophysiological basis of microvascular dysfunction in diabetes and summarize new interventions and their doses for managing microvascular dysfunction in diabetes.

## 2. Diagnosis of Microvascular Dysfunction in Diabetes

An effective preventive and management program of diabetes-associated microvascular dysfunction is grounded on the understanding of pathophysiological changes in prediabetes and diabetes that lead to the development of microvascular dysfunction [1,5,6]. To achieve this goal, imaging technologies with computational methods are needed to quantitatively assess microvascular dysfunction [11,12]. Optical and imaging technologies that provide in vivo noninvasive quantitative assessments of microcirculation are necessary for the screening and diagnosis of microvascular dysfunction and monitoring of diabetes progression. Recent advances in technologies with computational methods enable clinicians to provide the individualized management of microvascular dysfunction in diabetes. This section reviews imaging technologies and computational methods used to diagnose microvascular dysfunction. The summary of the major findings and common commercial brands of technologies as well as computational methods are listed in Table 1. The inclusion of studies is based on the search of Medline and IEEE databases.

### 2.1. Laser Doppler Flowmetry and Imaging

Laser Doppler technology is a common optical device used to monitor microcirculation and is based on using the Doppler Effect described by Johan Christian Doppler (1803–1853) to non-invasively assess microcirculation [13]. The Doppler Effect refers to a shift in the frequency of the sound when there is relative motion between the sound source and its observer. For laser Doppler devices, laser light is usually used as a source to monitor changes in the velocity of red blood cells. Specifically, laser light emitted by the laser Doppler device hits moving red blood cells and undergoes a frequency shift that is proportional to the velocity of the moving blood cells. The magnitude and frequency distribution of the scattered laser light can be detected by a receiver and can be used to estimate the red blood cell velocity. The measurement depth of a laser Doppler flowmeter is dependent on the measured tissue property, the wavelength of the laser light, and the distance between the light emitter and receiver in the laser Doppler probe [14]. The penetration depth of the commercial laser Doppler flowmetry devices is usually about 1 to 2 mm, which is sufficient to monitor cutaneous microcirculation [15]. The performance of laser Doppler is similar to other measures of tissue perfusion including isotropic clearance [15]. The current method of laser Doppler technology requires the patient to avoid movement during the assessment and cannot be used during exercise.

Two types of laser Doppler technologies are developed including laser Doppler flowmetry (LDF) and laser Doppler imaging (LDI) [11,12,16]. Laser Doppler technologies can be applied to monitor the microvascular function in various tissues and organs (e.g., the kidney, bone and skin) invasively and non-invasively. The advantage of LDI is to assess the skin blood flow over a large area, and the advantage of using LDF is to continuously measure dynamic changes in microcirculation reflecting vasomotion (rhythmic contractions of arterioles) over a small area [17]. Vasomotion reflects the overall regulation of the microvascular system to overcome the stimulus by modulating the vessel diameter and oscillation frequency in response to various stimuli for maintaining a sufficient blood and nutrient supply [18]. Thus, the use of laser Doppler to quantify the frequency power of blood flow oscillations may quantify the pathophysiological changes of underlying blood flow control mechanisms and the progression of microvascular dysfunction in diabetes [4,19]. The LDF signals are usually sampled at 32 Hz for the subsequent computational analysis. During the measurement of LDF signals, patients are required to not move in order to avoid movement artifacts in the blood flow signals. Various experimental procedures have been proposed to diagnose various impairments of microvascular dysfunction including impaired reactive hyperemia under externally applied mechanical stress on the skin and impaired thermal hyperemia under local thermal stress [19,20].

### 2.2. Transcutaneous Oximetry

Transcutaneous oximetry (TcpO_2_) is a non-invasive tool to assess the severity of peripheral arterial disease and prognosis after amputation and chronic wound healing [21]. The development of TcpO_2_ can be dated back to 1972 when Huch et al. used the heated Clark electrodes to monitor TcpO_2_ [22]. The devices developed by the author were used in clinical practice in neonates and had been demonstrated to reliably measure the arterial partial pressure of oxygen (PaO_2_). The use of TcpO_2_ requires the heating of the skin and when local heating is not used, the TcpO_2_ measurement of the skin is less than 3.5 mmHg. The purpose of adding local heating is to increase the permeability of the skin to oxygen molecules. The TcpO_2_ probe usually heats the skin to 44 °C for 15–20 min. The oxygen consumption in epidermal cells is very low and oxygen dissociation in the capillary network is very high in the healthy skin. Thus, measured TcpO_2_ at 44 °C could represent the arterial oxygen tension, while TcpO_2_ measured on unheated skin can be used to estimate the skin blood flow.

A normal range of TcpO_2_ is over 60 mmHg and when TcpO_2_ is at 20–30 mmHg or less in peripheral microcirculation, this indicates severe tissue ischemia [22]. It is reported that when TcpO_2_ reaches 0 mmHg, the oxygen is not zero, but there is a balance between the oxygen delivery and metabolic consumption of the skin. To date, no clinical organizations have endorsed the threshold value of TcpO_2_ for defining tissue ischemia and a need for vascular surgery or limb amputation. Therefore, clinicians will need to consider many factors including TcpO_2_ to diagnose microvascular dysfunction. Nevertheless, a timely vascular surgery may reduce the risk of limb amputation and healthcare costs in people with diabetes. The use of both laser Doppler and transcutaneous oximetry allows a comprehensive assessment of microvascular function in terms of the blood flow and oxygen supply.

### 2.3. Near-Infrared Spectroscopy

Near-infrared spectroscopy (NIRS) is a noninvasive, optical device using near-infrared light between 700 and 1000 nm to measure the concentration of oxyhemoglobin, deoxy-hemoglobin and myoglobin in the tissue. The major application of NIRS is on cerebral research and is starting to investigate exercise physiology and tissue ischemia [23,24]. The device is based on the Modified Beer–Lambert Law [24]. There are three types of NIRS oximeters including (1) a continuous wave, (2) time-domain or time-resolved spectroscopy and (3) frequency domain techniques. The most common type is continuous wave NIRS which uses the baseline value to calculate the change in the concentration of oxyhemoglobin and deoxy-hemoglobin. While time-domain and frequency-domain spectroscopy provide the absolute value of the hemoglobin concentration, continuous wave spectroscopy is much easier to operate and has a much lower cost and so is becoming the major choice for clinical practice.

The detection depth is dependent on the configuration of the wavelength of the emitting light and detector and can range from 12 (brain) to 120 (breast tissue) mm [23,24]. NIRS can also be used to investigate the muscle oxygen metabolism after exercise and the first study was conducted in the early 1990s [25]. This technology can be used to assess the hemodynamic response of the muscle to various stimuli in patients with DM. The sensor of NIRS devices is classified as a single sensor and a sensor array (e.g., 16 channels). The single sensor of NIRS usually has a higher sampling rate to characterize the time-series dynamics of the muscle hemodynamics while the sensor array of NIRS can be used to study the spatial distributions of hemodynamics and to overcome spatial variations [26].

### 2.4. Infrared Thermography

While LDF, TcpO_2_ and NIRS provide a real-time assessment of microcirculation, the cost of these devices prevent them from being widely used at home for long-term monitoring. Therefore, researchers have been identifying appropriate surrogates for long-term patient monitoring. Infrared thermography has been proposed to achieve this goal because of its low-cost and easy-to-operate features. Research studies have demonstrated that the skin temperature, measured through thermography, could be used to predict changes in the skin blood flow [27,28]. However, it requires further research to establish the relationship between the skin temperature and skin blood flow under various conditions, including various degrees of impairment and disease progression of diabetes and neuropathy. Nevertheless, infrared thermography is an affordable tool to indirectly estimate microcirculation for home use. In recent studies, the temperature difference over +2.2 °C on both plantar feet has been shown to be an effective surrogate measure for the early detection of diabetic foot ulcers [1,29]. The sampling frequency of NIRS signals ranges from 2 to 20 Hz. Although infrared thermography demonstrates its promise in assessing microvascular changes, further research is needed to establish the relationship between the skin temperature, blood flow and oxygenation.

### 2.5. Endothelial Assessment

The approach to assess endothelial function is to induce a stimulus (either infusion of endothelial-dependent vasodilators or reactive hyperemia) for assessing its regulation. Commonly used techniques for assessing endothelial function can be classified into two categories including conduit arteries and microvascular arterioles [30]. The conduit artery beds include coronary epicardial vasoreactivity (gold standard) and flow-mediated dilation (FMD) for the brachial conduit artery (much easier access and lower cost compared to coronary epicardial vasoreactivity). Microvascular beds include coronary microvascular function with Doppler wires, venous occlusion plethysmography and EndoPAT. Endothelium-dependent pharmacological triggers for microcirculation are Acetylcholine, Salbutamol and Bradykinin and endothelium-independent pharmacological triggers are Adenosine, Dipyridamole and Nitroprusside. FMD is the most widely used assessment for assessing endothelial function [31]. The technique measures the ability of the artery to responds to the stimulus (5 min occlusion of the brachial artery with a blood pressure cuff) by releasing nitric oxide. The peripheral endothelial function assessed by FMD correlated with the coronary artery endothelial function [30,31,32]. Villano investigated whether endothelial dysfunction is associated with an increased risk of cardiovascular disease and found that the endothelial function assessed by FMD might be used to identify patients at higher risk of cardiovascular events [33]. Therefore, the FMD assessment has been used as an index to assess the risk for cardiovascular disease in diabetes.

### 2.6. Computational Analysis of Microvascular Dynamics

Patterns of microvascular dynamics have been used to characterize the normal and abnormal microvascular regulation, including diabetes [5,19]. The blood flow in smaller vessels (arterioles) is well known for oscillatory patterns as opposed to steady ones. Vasomotion is a powerful approach to regulate the blood flow resistance and could be explained by a mathematical calculation. Here, two scenarios are discussed including one with a time-varying radius of the blood vessel and the other with a constant radius of the blood vessel. Nobel Laureate August Krogh found the heterogeneity of the blood flow in the webbed feet of frogs, and reported that the blood flow in capillaries may change the velocity periodically in the 1920s [34]. Later, research studies demonstrated that the measurements of the red blood cell velocity in individual capillaries exhibited fluctuations [35]. Griffith et al. indicated that spontaneous fluctuations in the radius of small blood vessels contributed to the nonlinear rheological properties of the blood flow [36]. Findings from numerous studies suggest that blood flow oscillations can be used to study microvascular regulation based on vasomotion [37,38]. Therefore, microvascular dynamics provides a new window to investigate microvascular dysfunction and complications in diabetes [4,19].

Skin blood flow signals measured by LDF and NIRS can be analyzed using linear, nonlinear and machine learning approaches to characterize the pathophysiological and adaptation of the microvascular system in prediabetes and diabetes [4,39]. Common analyses of microvascular dynamics include a time domain analysis (thermal index), frequency domain analysis (wavelet transform and wavelet-phase coherence analysis), nonlinear methods (sample entropy, fractal and correlation dimension) and the machine learning of artificial intelligence (e.g., AlexNet, Convolutional Neural Network (CNN), GoogLeNet, Vgg-19 and ResNet-18) [39]. The linear analysis is usually used to quantify the overall microvascular regulation in response to a stimulus, for example, the thermal index and power of a frequency band related to a blood flow control mechanism. The nonlinear analysis can quantify the influences and interactions of the control mechanisms of blood flow oscillations that cannot be detected in the linear method [18,40,41]. The use of both linear and nonlinear methods would complement each other to better understand microvascular regulation and dysfunction in diabetes.

The wavelet transform (linear spectral analysis) of blood flow oscillations is a relatively new method and has attracted attention from microcirculation researchers for its potential to noninvasively monitor several blood flow regulatory mechanisms. The method was first reported by Anita Stefanovska in 1999 for six characteristic frequencies embedded in blood flow oscillations reflecting the influence of heart rate (0.4–2 Hz), respiratory activity (0.15–0.4 Hz), vascular myogenic activity (0.05–0.15 Hz), neurogenic activity (0.02–0.05 Hz) and two metabolic endothelial controls (nitric-oxide-dependent and nitric-oxide-independent between 0.005 and 0.02 H) [40]. The power within each frequency band has been used to characterize the activity of the corresponded mechanism, with a larger power (wavelet amplitude) indicating an active regulation from the related control mechanism [18,40,41]. The microvascular dynamics assessed by LDF and NIRS has shown promise to diagnose the underlying impairment of microvascular dysfunction related to the metabolic endothelial, neurogenic and myogenic controls, while the exact meanings of these characteristic frequencies in blood flow oscillations remain to be determined [5]. Nevertheless, these computational methods can be used to characterize complex changes associated with microvascular dysfunction in diabetes.

**Table 1:** Major findings of diagnosis and management of microvascular dysfunction in diabetes.

Diagnosis	**Technology/Methods**	**Principle and Representative Devices**	**Pros**	**Cons**
	Laser Doppler Flowmetry and Laser Doppler Imaging [19]	The device is based on Doppler effect to use low-level laser (1–2 mW) to monitor blood flow velocity in perfusion unit. Major brands include Perimed PeriFlux 6000 and Moor moorVMS-LDF.	- Non-invasive, real-time monitoring. - Can be combined with a variety of computational methods for advanced analysis. - Can be combined with iontophoresis for endothelial assessment.	- Higher sampling frequency (e.g., 32 Hz) is only allowed to laser Doppler flowmetry. - Larger sampling area is only allowed by laser Doppler imaging with a low sampling frequency (e.g., 2 Hz).
	Transcutaneous Oximetry [14]	The device uses local heating to measure oxygen (O_2_) and carbon dioxide (CO_2_) molecules in mmHg. Major brands include Perimed tcpO_2_ and Radiometer TCM.	- Can monitor the oxygen level (rather than blood flow).	- Local heating is needed. - A longer duration of assessment is needed (e.g., 20 min) for allowing thermal vasodilation.
	Infrared Thermography [42]	The device can measure the skin temperature in °F. Major brands include FLIR Systems E6-XT.	- The most affordable device for home-use and point-of-care facilities.	- An indirect measure of microvascular function.
	Near-infrared spectroscopy [23]	The use of Beer–Lambert Law to assess the concentration of hemoglobin in the tissue in μM. A number of brands are in market including Hamamatsu, Hitachi, NIRx and ISS.	- Can be used to assess oxyhemoglobin, deoxy-hemoglobin, total hemoglobin and oxygenation. - Can be used to assess muscle hemodynamics.	- The operation of NIRS requires extensive training.
	Computational Methods [19,39]	The use of signal and imaging processing to classify microvascular status, including linear (thermal index, wavelet), nonlinear (sample entropy, fractal, correlation dimension, wavelet phase coherence) and machine learning (convolutional neural network, AlexNet, Vgg-19, GoogLeNet, ResNet-18).	- Various computational methods, including time domain, frequency domain, nonlinear domain, and machine learning approaches, can be used to assess the progression of diabetes.	- High computing power is needed for complex analysis. - The clinicians need numerical analysis and programming training.
Intervention	**Modality**	**Principle**	**Benefits**	**Guidelines (Dosage)**
	Weight-bearing exercise [9,10,43,44]	Weight-bearing exercise is recommended to people with diabetes, including people at risk for diabetic foot ulcers.	- Activities of daily living can be integrated for exercise programs. - Weight-bearing activities stimulate plantar tissues to maintain sufficient stiffness.	- Both aerobic and resistance exercise [10]. - Moderate-to-rigorous intensity of exercise for 150 min per week [43]. - People who are physically inactive should start from breaking down sedentary time before pursuing exercise [9]. - Both upper and lower limb muscles [44].
	Non-weight-bearing exercise [9]	Non-weight-bearing exercise is needed for people with open diabetic foot ulcers.	- Non-weight-bearing activities may reduce risk for diabetic foot ulcers.	- With diabetic foot ulcers, non-weight-bearing exercise can promote wound healing.
	Local Vibration [45]	The use of vibration to stimulate blood flow control for improving microcirculation.	- Mechanical vibration improves blood flow to reduce tissue ischemia. - Patients do not need to exercise.	- Intermittent local vibration can be applied to plantar foot.
	Thermal Therapy [46]	The use of heat to induce a vasodilatory response for improving microcirculation.	- Thermal therapy improves blood flow to reduce tissue ischemia. - Patients do not need to exercise.	- Local heat like warm bath could induce thermal vasodilation for improving microcirculation. A careful assessment is needed to avoid overheating in people with diabetic neuropathy.

## 3. Pathophysiology of Microvascular Dysfunction in Diabetes

Microvascular dysfunction has been demonstrated to be associated with the progression in prediabetes and diabetes [1,46,47]. Under pathophysiological changes of diabetes [7], structural changes and functional adaptations of the microvascular system can be assessed using optical and imaging technologies with computational methods reviewed in the previous section. Clinically, microvascular dysfunction is usually diagnosed as an impaired hyperemic response to a stimulus reflecting as reactive hyperemia, thermal hyperemia and exercise hyperemia [1,48]. This is because the microvascular function is featured for temporal and spatial variations and the degree of impairment is better quantified through a response to a stimulus. This section reviews the microvasculature, microvascular regulation and microvascular dysfunction in diabetes.

### 3.1. Microvasculature

The cutaneous microvascular system is the model system to study the normal and pathological regulation of microcirculation for its ease of access in diabetes. The microvascular system consists of blood vessels with a diameter of less than 150 μm. The majority of the skin blood flow (~85%) is used to regulate body temperature, and a smaller part of the skin blood flow (~15%) is used for the metabolic purpose. Under thermal stress (hot weather), the skin blood flow increases to remove the excessive heat from the body and under cooling (in cold winter weather), a vasoconstrictive response, occurs to limit the blood flow to the skin to avoid the loss of heat from the body core to the environment. As discussed in Section 2.5, the body uses the vasomotion to quickly adjust the blood flow to the skin. The vasculature of the skin consists of two interconnected systems including the superficial vascular plexus in the dermis and the deep vascular plexus at the dermal–subcutaneous interface [15]. The two vascular plexuses are connected by paired ascending arterioles and descending venules [15].

Cutaneous microcirculation has been investigated to characterize early changes associated with diabetes. Also, skin microcirculation can be used to estimate the status of other microvascular systems [49]. This characteristic is particularly important because diabetes can cause generalized microvascular dysfunction in various organs and some of them may not be easily assessed. The skin is the most accessible organ and plays a major role in many body function, such as thermoregulation and water homeostasis [50]. Two types of the skin, glabrous skin and non-glabrous (hairy) skin exist on our body. Glabrous skin contains a large number of highly innervated arteriovenous shunts and plays a major role in thermoregulation. Blood vessels in the skin consist of arterioles, arterial and venous capillaries and venules. The arterioles where vasomotion occur serve as a critical part of the resistance vessels for regulating the distribution of microcirculation [15,51]. In the nonglabrous skin, blood vessels are innervated by both adrenergic and sympathetic cholinergic nerves, and blood vessels in the glabrous skin are regulated by sympathetic adrenergic nerves. These differences indicate that potential interventions for managing microvascular dysfunction should be assessed in both glabrous and non-glabrous skin.

### 3.2. Microvascular Regulation

Microvascular regulation is used to overcome the influences induced by a stimulus (either an environmental stress or pathophysiological condition) by regulating the blood flow to various organs and tissues. The regulation is through the change in vascular resistance for redistributing the blood flow [52]. Small blood vessels, specifically arterioles, can change their diameter by altering the contractile state of smooth muscles in the vascular wall. The change in the diameter of the arteriole leading to changes in vascular resistance can be determined as an approximately inverse proportion to the fourth power in the vessel diameter [52]. Thus, a small increase in the diameter would lead to a large increase in the blood flow. This statement can be further supported by a computational comparison between two scenarios.

The blood flow of the blood vessel with a constant radius (*r*_1_) can be computed as (Equation (1)): if $r_{1}=constant=r_{0}$

(1) $$\mathrm{Then}\;F_{1}=\frac{\Delta P}{R}=\frac{\Delta P}{\frac{8\mu L}{\pi r_{1}^{4}}}=\frac{\Delta P\cdot \pi r_{1}^{4}}{8\mu L}(\mathrm{according}\;\mathrm{to}\;\mathrm{Flow}=\frac{\mathrm{Pressure}\;\mathrm{Gradient}}{\mathrm{Resistance}});$$

where ΔP is the pressure gradient over the length *L* of a cylindrical vessel with the radius *r* and μ is the viscosity of the blood flow. 

The blood flow of the blood vessel with a time-varying radius (*r*_2_) can be computed as (Equation (2))*:* if $r_{2}=r(t)=r_{0}(1+\lambda \sin\omega t)$ (let the mean radius of two conditions be the same) and 0<λ<1 (assume a sinusoidal wave pattern of the vasomotion), then

(2) $$F_{2}=\frac{\int_{0}^{T}F(t)dt}{T}=\frac{\omega }{2\pi }\int_{0}^{2\pi /\omega }F(t)dt\;=\frac{\omega }{2\pi }\int_{0}^{2\pi /\omega }\frac{\Delta P\cdot \pi \cdot r_{0}^{4}\cdot {\left(1+\lambda \sin\omega t\right)}^{4}}{8\mu L}dt\;=\frac{\omega \cdot \Delta P\cdot \pi \cdot r_{0}^{4}}{16\pi \cdot \mu \cdot L}{\int_{0}^{\frac{2\pi }{\omega }}\left(1+\lambda \sin\omega t\right)}^{4}dt\;=\frac{\pi \cdot \Delta P}{8\mu \cdot L}(1+3\lambda^{2}+\frac{3}{8}\lambda^{4})\cdot r_{0}^{4}$$

By comparing the blood flow between the two scenarios (*F*′), the effect of a time-varying radius on the skin blood flow can be computed as (Equation (3)):

$$\mathrm{Normalized}\;\mathrm{blood}\;\mathrm{flow}\;=\frac{\mathrm{Flow}\;\mathrm{at}\;\mathrm{time}\text{-}\mathrm{varying}\;\mathrm{radius}\;\mathrm{of}\;\mathrm{the}\;\mathrm{vessel}}{\mathrm{Flow}\;\mathrm{at}\;\mathrm{constant}\;\mathrm{radius}\;\mathrm{of}\;\mathrm{the}\;\mathrm{vessel}}$$

(3) $$F^{\prime }=\frac{F_{2}}{F_{1}}=1+3\lambda^{2}+\frac{3}{8}\lambda^{4}$$

Equation (3) indicates that when the amplitude of the oscillation is larger than 0 (the amplitude of vasomotion), the blood flow increases, and when the amplitude is larger, the increase in blood flow is larger.

Reactive hyperemia refers to the increase in blood flow following the release of a compression force via blood-pressure-cuff-induced arterial occlusion or a directly applied mechanical force [53]. Reactive hyperemia is featured for an initial peak in the blood flow followed by a sustained increase in the blood flow. This vasodilatory response has been used to assess endothelial dysfunction and changes in the microvascular function following pharmacological intervention [54]. Although this assessment is widely used in clinical practices, the exact mechanisms responsible for reactive hyperemia remain unclear. Research studies suggest that reactive hyperemia is associated with endothelial vasodilators and myogenic responses [55,56].

Thermal regulation is a major role of cutaneous microcirculation for maintaining the thermal homeostasis of the body. The application of local heating to the skin induces a vasodilatory response and such an increase in the skin blood flow is dependent on the degree (e.g., 40 or 44 °C) and rate (fast or slow) of heating [18,57]. When the skin is under a fast heating protocol to 42 °C, a biphasic vasodilatory response is usually observed, including an initial peak blood flow (first peak), followed by a decrease to a lower value (valley) and then a moderate increase to a prolonged plateau phase (second peak/plateau). The initial vasodilatory response is mediated by a local sensory reflex and the second plateau is mediated by nitric oxide [58]. There is also evidence suggesting that the sympathetic neurotransmitters norepinephrine is involved in both the initial peak and second plateau phases [59]. The local cooling of the skin typically causes an initial decrease in the skin blood flow followed by a transient vasodilation and a progressive vasoconstriction [60,61]. The rate of change in the temperature and the extent of the temperature change can influence the blood flow response [62]. The vasoconstrictive response induced by local cooling is through the inhibition of the release of nitric oxide [63], in part at the level of the nitric oxide synthase enzyme and in part at steps downstream from the production of nitric oxide [60].

### 3.3. Microvascular Dysfunction in Diabetes

The epidemiology data indicate that hyperglycemia is associated with cardiovascular disease in people with diabetes. Clinicians have been exploring whether the reduction in glycemia would lead to a decreased risk for cardiovascular disease. However, clinical trials, including ACCORD in 10,251 participants [64] and ADVANCE in 11,140 participants [65], demonstrated that intensive glycemic control targeting HbA_1c_ could not reduce cardiovascular complications in diabetes [66]. These clinical trials also indicate that the hyperglycemic condition is remembered in the vascular system (i.e., a long-term effect of hyperglycemia even after glycemia has returned to normal). Therefore, the management of inflammation and oxidative stress and lifestyle modifications are more effective interventions for reducing the risk for cardiovascular disease, including microvascular dysfunction.

Diabetes causes macrovascular and microvascular complications. Macrovascular complications include peripheral artery disease, cardiovascular disease and cerebrovascular disease and microvascular complications include diabetic retinopathy, diabetic nephropathy, diabetic neuropathy and the microvascular dysfunction of various organs and tissues. Vascular endothelial dysfunction has been recognized as a key factor contributing to microvascular and macrovascular disease in diabetes [67]. Endothelial dysfunction is characterized by decreased endothelium-dependent vasodilation, chronic inflammation, and hyper-permeability. Four major factors associated with diabetes including hyperglycemia, hyperlipidemia, adipokines and insulin resistance induce oxidative stress for a reduction in the nitric oxide bioavailability of endothelial cells. Costantino et al. investigated whether the epigenetic regulation of the adaptor protein p66^Shc^, a key driver of mitochondrial oxidative stress, contributes to persistent vascular dysfunction [68]. The results demonstrated that glucose fluctuations contribute to chromatin remodeling and may explain persistent vascular dysfunction in people with type 2 diabetes with target HbA_1c_. A comprehensive review of molecular mechanisms and pathways underlying vascular complications of diabetes is provided by Paul and colleagues, who discuss the diabetes-induced activation of biochemical pathways and the related consequential four pathways [69]. Kaze et al. further explored the association between microvascular disease and cardiorespiratory fitness in diabetes and found that the presence of microvascular disease and its burden significantly correlates with cardiorespiratory fitness. This study underscores the importance of addressing microvascular disease in diabetes for improving cardiorespiratory fitness and reducing diabetes-related morbidity and mortality [70].

Laser Doppler technology has been used to screen and assess early microvascular dysfunction in diabetes [71]. Although the exact mechanisms responsible for microvascular dysfunction in diabetes have not been established, it is widely accepted that several causative factors are related, including a loss of neurogenic vasodilation [72,73] and endothelial nitric oxide synthesis [74]. These impairments can be quantified through a stimulus–response assessment (e.g., thermal vasodilation) using LDF and NIRS in people with diabetes [71]. Post-occlusive reactive hyperemia is another common test on assessing microvascular function [71,75,76]. Jan and colleagues used the wavelet analysis of LDF signals to assess the microvascular response to local thermal and mechanical stresses on the plantar foot in people with diabetic peripheral neuropathy and found that patients with diabetic peripheral neuropathy have significantly decreased metabolic endothelial, neurogenic and myogenic responses to thermal stress [71]. Furthermore, the authors also demonstrated an impaired blood flow oscillation pattern and suggested that skin blood flow oscillations in response to external stresses can be used to screen risk for microvascular complications, such as diabetic foot ulcers and retinopathy [71].

The studies demonstrate promising evidence that skin microcirculation can be used as a surrogate to assess the microcirculation of different organs and tissues (e.g., kidney, eye, and coronary microcirculation) because of the generalized feature of the microvascular systems of the body. Jan et al. applied wavelet analysis and sample entropy analysis to quantify the linear and nonlinear characteristics of microcirculation and found that people with diabetic peripheral neuropathy had a higher skin blood flow and higher degree of regularity on the planar foot compared to the dorsal foot and people with diabetic peripheral neuropathy had a higher plantar and dorsal skin blood flow compared to the healthy controls [19]. Zhao et al. demonstrated that impaired microvascular dynamics is associated with hyperglycemia in prediabetes and diabetes in the Maastricht Study reported in 2024 [4]. This finding further confirms the feasibility of using microvascular dynamics (skin blood flow oscillation patterns) for the screening and early diagnosis of prediabetes and diabetes.

## 4. Management of Microvascular Dysfunction in Diabetes

Since the pathogenesis of microvascular dysfunction in diabetes is not fully understood, the management strategy of microvascular dysfunction is centered on the glycemic control and lifestyle modification and physical activity (i.e., the current guidelines used to manage diabetes) [4,43,77]. ADA recommends both aerobic and resistance exercise programs for managing hyperglycemia [10,78]. Recent findings are discussed and evaluated in this section. The summary of major findings and treatment guidelines are provided in Table 1. The search strategy is to cover all types of interventions for improving the microvascular function in diabetes from PubMed.

### 4.1. Physical Activity and Exercise

Physical activity is an effective intervention to regulate blood glucose for slowing the progression of microvascular dysfunction in diabetes [43,79]. Physical activity and exercise programs have demonstrated promise on lowering hyperglycemia and improving macrovascular function in a meta-analysis, while the effect of exercise on microvascular function is largely unknown [10,78]. In earlier years, excessive weight-bearing exercise has been classified as a risk factor for foot ulcers in people with DM; as a result, weight-bearing exercise was not recommended for people with diabetes [80,81,82,83]. The current guidelines of physical activity from ADA suggest that people with type 2 diabetes should exercise in three or more sessions per week and the intensity should be moderate intensity (e.g., brisk walking) or high intensity; the total exercise time of a week needs to be over 150 min and 2 or more continuous days without exercise should be avoided [43,84]. Both upper limb exercise and lower limb exercise should be incorporated into the exercise program [44]. An appropriate physical activity intensity for diabetes control and microvascular function remains to be determined. A consensus has been reached that an effective exercise program would greatly slow down the progression of microvascular dysfunction and its impact on diabetic foot ulcers, muscle fatigue and weakness and peripheral neuropathy. However, it is imperative to determine the dose–response relationship of exercise and microvascular responses in patients with diabetes. Ren and colleagues investigated the effect of the exercise volume on plantar microcirculation and tissue hardness in people with diabetes and found that the high exercise volume group had significantly higher oxygen saturation and significantly lower hardness of the planar tissue compared to the low exercise volume group [85]. The author’s results provide the first evidence that the exercise volume is associated with plantar microcirculation and supports that weight-bearing exercise may not increase the risk for diabetic foot ulcers. Crews et al. critically evaluated the available literature on the perception of exercise in people with diabetes and discussed the challenges of increasing physical activity and reducing the sedentary time in people with diabetes [9].

### 4.2. Force-Based Interventions

Research studies have demonstrated that local vibration and whole-body vibration can improve microcirculation in various pathological conditions, including diabetes. Ren et al. demonstrated that local intermittent vibration (50 Hz, 2 mm amplitude, 10 s vibration and 5 s rest for a total of 7.5 min; 10 s vibration and 10 s rest for a total of 10 min) can effectively increase the skin blood flow, but not the local continuous vibration (50 Hz, 2 mm amplitude, 5 min) of the foot in people with diabetes [45]. In another study conducted by the same group, the authors explored the effect of intermittent pneumatic compression with different inflation pressures on the skin blood flow of the foot and found that 120 mmHg was effective in improving the skin blood flow of the foot [86]. When a patient has diabetic foot ulcers, an evaluation is needed to determine whether the patient should continue pursuing exercise [87].

### 4.3. Thermal Stress-Based Interventions

Thermotherapy is an adjunct treatment of microvascular dysfunction and diabetic foot ulcers because thermal stress can induce vasodilatory responses for improving microcirculation [42,88]. Thermal modalities are commonly used to increase the local blood flow, which is thought to contribute to reduce tissue ischemia and improve wound healing [89]. Thermal modalities include superficial heat (e.g., infrared thermal treatment) and deep heat (ultrasound treatment) [90]. Due to the equipment cost for the aforementioned thermal modalities, alternative forms of passive heat are usually used including sauna and hot-tub bathing. Although thermal interventions have demonstrated their promise in improving cardiometabolic outcomes, the evidence remains insufficient.

## 5. Future Directions

Due to the complex nature of microvascular regulation, various computational methods have been developed to shed light on the influence of diabetes on microvascular dysfunction. However, microcirculation reflects a complex interaction in the microvascular system consisting of networks of arterioles, capillaries and venules and the causal relationships between various pathophysiological factors on the genesis of microvascular dysfunction remain challenging [52]. As a result, computational methods have not been applied to screen diabetes and pre-diabetes based on microvascular dynamics. The emerging of the machine learning and deep learning of artificial intelligence (AI) may assist the clinicians by developing smart algorithms to diagnose microvascular dysfunction [39,91]. The development of an AI-based model requires extensive data for training, classification and validation, which will require extensive resources, including computing power and manpower to develop such smart algorithms [92]. However, the “hidden layer” of the neural network used to classify and predict microvascular dysfunction may be a feasible solution before the mechanism of microvascular dysfunction in diabetes has been developed.

As there is no disease-modifying therapy [93], physical activity and lifestyle modifications are recommended for the management of diabetes-related complications, including microvascular dysfunction and diabetic neuropathy [10,93]. The existing literature focuses on the effects of exercise training on managing hyperglycemia and macrovascular function and ignores the influences on microvascular dysfunction and diabetic neuropathy. A systematic review and meta-analysis indicated that exercise training is an effective intervention in reducing hyperglycemia in people with diabetes, and combined aerobic and resistance exercise is more effective than aerobic or resistance exercise alone. Exercise also has a significant effect on improving the ankle brachial index, suggesting that it can play a role in the prevention of peripheral arterial disease (macrovascular function). However, evidence regarding the association between exercise and improvement in microvascular dysfunction has not been well investigated. Future research is needed to clarify the effectiveness of exercise in terms of various modes (aerobic vs. resistance exercise, upper vs. lower limb exercise, and weight bearing vs. non-weight bearing exercise) on improving the microvascular function in the glabrous and nonglabrous skin in patients with diabetes.

The prevalence of type 2 diabetes is estimated at 415 million people worldwide and is expected to rise to 642 million by 2040 [68]. Among the diabetes-related comorbidities, cardiovascular disease is among the most significant. A study developed a cost of diabetes economic model to estimate the economic burden of diabetes and the estimated cost of diagnosed diabetes in the United States in 2022 was USD 412.9 billion [94]. A systematic review indicates that the specific cost related to managing cardiovascular disease in diabetes is not well documented. Thus, the economic burden of macrovascular and microvascular complications should be investigated to better understand the burden of diabetes complications [95]. Nevertheless, the impact of diabetes-related costs is enormous; therefore, any early diagnosis and management would have a significant impact on the healthcare system and economics.

This is a comprehensive review aiming to analyzing relevant broad topics related to microvascular dysfunction in diabetes ranging from diagnosing technologies/techniques, pathophysiological bases and interventions. The search strategy was to cover all types of techniques and interventions using the studies written in English and published in PubMed and IEEE databases. Future studies may need to perform a systematic review to cover all relevant studies to minimize the selection bias. Furthermore, most of the intervention studies discussed in this review had study design limitations, including a small sample size, a lack of a control group and a lack of long-term-effect monitoring. Future studies should consider these limitations and conduct larger clinical trials to validate these findings. Last, many technologies and techniques discussed in this review have not been systematically investigated in terms of their sensitivity, reliability and specificity on diagnosing microvascular dysfunction and comparisons of established biochemical markers. These issues should be addressed before clinical practice using their new technologies is carried out.

## 6. Conclusions

In this comprehensive review paper, recent advances in imaging and optical technologies with computational methods used to diagnose microvascular dysfunction are reviewed. The pathophysiology of diabetes-associated microvascular dysfunction is discussed and the effectiveness of interventions on managing microvascular dysfunction is evaluated. The major findings from this comprehensive review are summarized to benefit clinicians to evaluate these new methods and guidelines. Most technologies are commercially available. An advanced computational analysis of blood flow oscillations requires basic computational and programming skills of the clinicians to implement them. The development of a user-friendly interface may be needed for the application of these computational methods in clinical practice. The exercise guidelines are important and should be conveyed by clinicians to patients with diabetes for taking both aerobic and resistance exercise in upper and lower limbs for 150 min per week as well as to periodically break down sedentary activities. The use of microvascular dynamics based on linear, nonlinear and machine learning approaches is recommended for the screening and early diagnosis of diabetes.
